# Investigation of age-related facial variation among Angelman syndrome patients

**DOI:** 10.1038/s41598-021-99944-z

**Published:** 2021-10-21

**Authors:** Olalekan Agbolade, Azree Nazri, Razali Yaakob, Abdul Azim Ghani, Yoke Kqueen Cheah

**Affiliations:** 1 Department of Computer Science, Faculty of Computer Science and IT, Universiti Putra Malaysia, Serdang, Selangor Darul Ehsan Malaysia; 2 Department of Software Engineering, Faculty of Computer Science and IT, Universiti Putra Malaysia, Serdang, Selangor Darul Ehsan Malaysia; 3 Department of Biomedical Science, Faculty of Medicine and Health Sciences, Universiti Putra Malaysia, Serdang, Selangor Darul Ehsan Malaysia

**Keywords:** Developmental biology, Ageing, Computer science

## Abstract

Angelman syndrome (AS) is one of the common genetic disorders that could emerge either from a 15q11–q13 deletion or paternal uniparental disomy (UPD) or imprinting or UBE3A mutations. AS comes with various behavioral and phenotypic variability, but the acquisition of subjects for experiment and automating the landmarking process to characterize facial morphology for Angelman syndrome variation investigation are common challenges. By automatically detecting and annotating subject faces, we collected 83 landmarks and 10 anthropometric linear distances were measured from 17 selected anatomical landmarks to account for shape variability. Statistical analyses were performed on the extracted data to investigate facial variation in each age group. There is a correspondence in the results achieved by relative warp (RW) of the principal component (PC) and the thin-plate spline (TPS) interpolation. The group is highly discriminated and the pattern of shape variability is higher in children than other groups when judged by the anthropometric measurement and principal component.

## Introduction

Angelman syndrome (AS: OMIM# 105830) is 1 in 12,000–20,000 of the population^1,2^ and characterized by speech impairment, developmental delay a unique behavior with a happy demeanor that includes frequent laughing, gait ataxia and/or tremulousness of the limbs, and excitability, seizures, and microcephaly^3,4^. Besides these, there are noticeable physical characteristics such as protruding tongue, occipital groove, flat occiput, widely spaced teeth, wide mouth, prognathia, frequent drooling, and strabismus^4–6^.

The clinical features of AS do not manifest until after age 1 year even though developmental delays are first noted at around 6 months, it may take several years before the correct clinical diagnosis can be made^5^. Since there may be no obvious dysmorphic features and the multiple genetic mechanisms that cause AS, timely diagnosis poses a challenge to the clinician. In suspecting the diagnosis, a behavioral phenotype is a crucial element^5^. Through a genetic study of AS, 15q11–q13 deletions are found in approximately 70–75% of individuals with AS which are of maternal origin^7,8^. Furthermore, paternal uniparental disomy (UPD) of chromosome 15 is also found in about 2–3% of the patients^6,8,9^.

In previous studies, clinical and behavioral manifestations of 4 cases of paternal UPD15 among Brazilian AS children were described in^10^. They compared their study to the UPD cases from the literature in^11,12^. There is also a phenotypic variability comparison by the same authors in^6^ by adding four new cases which showed an AS patient with paternal isodisomy by collecting three sources of data in^13^: literature review, physical examination, and questionnaire data of affected individuals. The authors experimented that individuals with and without a deletion could not be differentiated clinically. Concluding that diagnosis in early childhood is difficult and a high index of suspicion is recommended. Investigation of the relationship between age and smiling and laughing was proposed in^14^ on 24 AS children. The experiment was based on three conditions: restricted social interaction, proximity only, and social interaction. The results showed a decline in smiling and laughing in the oldest group. In^15^, ten subjects age from 5 to 11 years confirmed diagnosis of AS (3 UBE3A: OMIM# 601623 mutation and 7 15q11.2–q13 deletions). The evaluation was based on cognitive, adaptive, communication, behavioral and neurovisual aspects. Though these studies are theoretical, yet they presented important information regarding AS patients. However, they are all faced with the challenge of small sample size for proper experiment and analysis. Dataset has been a major challenge in the field of neurological study or genetic disorder analysis. The studies in^16–18^ applied morphometric approach to detect facial landmarks and analyze face morphology in genetic syndrome but the studies focused only on down syndrome patients, whereas the study in^19^ focused on the face morphology based on multiple genetic syndromes including AS; all with dataset collected from an internet source and they are not age-related. Thus, our study is based on the investigation of variation in facial analysis among AS patients based on age using a morphometric approach. Based on our studies, no such method has been applied to investigate the age-related variation of AS in morphometric which makes this study novel.

The landmark-based geometric morphometrics methods for face investigation provide new insights into patterns of biological shape variation that could not be evaluated by traditional methods^20^. Landmarks are points of correspondence on each object that matches within and between populations. This set of points, one on each form, that is operationally defined on each individual by local anatomical features must be consistent with some hypothesis of biological homology^21^. Geometric Morphometrics (GM) of landmarks have been used extensively for quantifying shape variation in biological subjects^22^ and frequently to examine shape variation in biometric fields. But automating the landmarking process to characterize morphological traits for developmental variation investigation has been very tasking. Below is the summary of the main contribution of this work:

1. Due to the nature of the dataset which suffers from different postures, occlusion, and expression. We employed automatic face detection and landmarking algorithm in^23^. This automatically detected the face regardless of posture or expression with 83 facial landmarks.
2. Among the 83 landmarks detected, 17 anatomical landmarks were selected covering the eye, nose, mouth, chin, and cheek regions which have shown robust performance in detecting shape differences in genetic disorders^24^, to perform anthropometric measurement for further facial analysis. These were visualized using relative warp (RW) of Principal Components Analysis (PCA) and thin-plate spline (TPS).
3. From the 17 anatomical landmarks, 10 inter-landmark linear distances were computed using Euclidean Distance Matrix Analysis (EDMA) on each age group to measure the variations in the selected regions. Then further statistical analyses were such as Principal Components Analysis (PCA), Canonical Variates Analysis (CVA), Multivariate Analysis of Variance (MANOVA), and Discriminant Function Analysis (DFA) were performed to arrive at conclusions.

**Figure 1:**
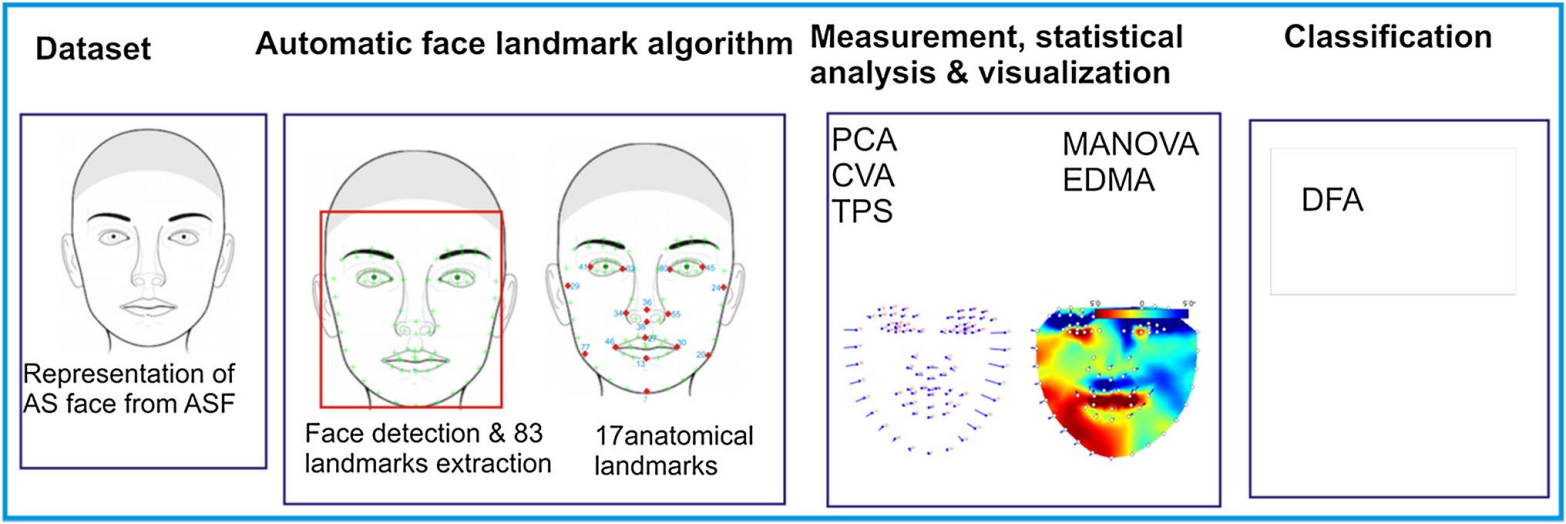
Architectural diagram for the proposed method.

The rest of the sections are organized thus: section two focuses on the materials and methods with supporting references where a short explanation has been provided. Section three presents the results and discussion of the implementation and concludes the study with the limitations and future direction. Figure 1 shows the architectural diagram of the step-by-step approach used in this study.

## Materials and methods

### Dataset and description

We collected 140 face images directly from Angelman Syndrome Foundation (ASF)^25^ with permission between 8 and 60 years of age, which are publicly available images of patients with AS submitted in PNG and JPG format. The following two exclusion criteria were applied: 1. The eyes and mouth regions needed to be visible for accurate detection by the algorithm. 2. There was a correct diagnosis confirmation inspection by an expert clinician to validate the supposed syndrome. All images that did not meet the criteria were discarded and only 116 images were finally used in the analyses. The age group in years is sub-divided into four categories: children (below 13 years): 24 , teenagers (13–19 years): 39, young adults (20–29 years): 33, and adults (30 years and above): 20.

### Geometric morphometric analysis

**Figure 2:**
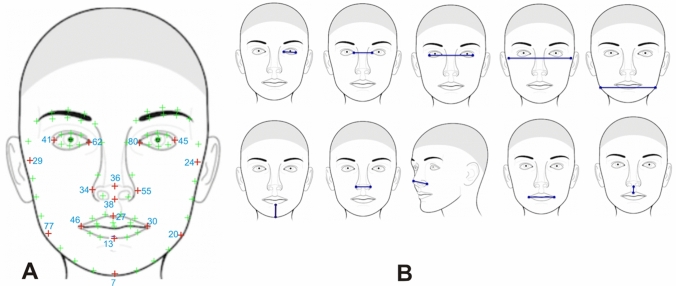
Facial landmarks and anthropometric measurements used in the study. (**A**) Shows an example of 83 automatically detected facial landmarks in green with the 17 anatomical landmarks in red while the number notation in light blue. (**B**) Approximate location of the 10 anthropometric linear distances used in the analysis redacted from^27^.

Due to various postures of the subjects, the face images were automatically detected and landmarked using the algorithm in^23^. Through the algorithm, 83 landmarks (details in supplementary info S1) were automatically annotated covering eyelid, eye, nose, mouth, chin, and cheek regions. To perform further measurement, 17 anatomical landmarks were selected. These landmarks were selected because they are visible on all specimens and are useful in detecting shape differences in genetic disorders^24^. Figure 2A shows the positions of the anatomical points; Fig. 2B shows the approximate location of the 10 anthropometric linear distances; while Table 1 shows the description of the anatomical landmarks. The raw landmarks were first subjected to a General Procrustes Analysis (GPA) which scales, centers, and rotates the entire set of landmark configurations so that they are aligned within a common coordinate system^26^.

**Table 1:** Anchor anatomical points and descriptions.

No.	Anatomical point	Description
41	Endocanthion left	Left most medial point of the palpebral fissure, at the inner commissure of the eye
62	Exocanthion left	Left most lateral point of the palpebral fissure, at the outer commissure of the eye
80	Exocanthion right	Right most lateral point of the palpebral fissure, at the outer commissure of the eye
45	Endocanthion right	Right most medial point of the palpebral fissure, at the inner commissure of the eye
36	Pronasale	The most anteriorly protruded point of the apex nasi
38	Subnasale	Median point at the junction between the lower border of the nasal septum and the philtrum area
34	Alare left	Left most lateral point on the nasal ala
55	Alare right	Right most lateral point on the nasal ala
46	Cheilion left	Left outer corners of the mouth where the outer edges of the upper and lower vermilions meet
30	Cheilion right	Right outer corners of the mouth where the outer edges of the upper and lower vermilions meet
27	Labiale superius	Midpoint of the vermilion border of the upper lip
13	Labiale inferius	Midpoint of the vermilion border of the lower lip
7	Gnathion	The lowest point in the midline on the lower border of the chin
77	Gonion left	The most lateral point at the angle of the mandible left
20	Gonion right	The most lateral point at the angle of the mandible right
29	Zygion left	The most lateral point on the zygomatic arch left
24	Zygion right	The most lateral point on the zygomatic arch right

Anthropometric Measurement was peformed using Euclidean Distance Matrix Analysis (EDMA)^28,29^. From the 17 selected anatomical landmarks, 10 inter-landmark distances based on standard anthropometric measurement in^27^ were computed for each group and we took the log of all distances to two decimal place. EDMA does not only provide an objective measurement of shape differences but also localizes the sites of major variations by suggesting which of the landmarks are more involved in the form difference^30^.

### PCA, CVA and TPS

After the GPA which computes the consensus configuration, the Principal Components Analysis (PCA) was used to explore the morphospaces which shows the distribution of the specimen. Multivariate Analysis of Variance (MANOVA) and Canonical Variates Analysis (CVA) were used to test significant differences between age groups. CVA differs from PCA in that it requires specimens to be assigned to the age group (pre-defined group), and then tests how well the scores can be used to support those assignments. It aims at maximizing the ratio of the between-group variance to the within-group variance. Axes are scaled according to patterns of within-group variation and are not simple rotations of the original coordinate system as in PCA^31^. The CVA was computed based on the first 50 PCs which accounted for 99% of the total shape variation in all ages group.

To visualise the facial variation in age group, lollipop graph of the first principal component was plotted, using the mean shape of the source configuration. This shows the shifts of landmark positions with straight lines. The length and direction of the line indicate the movement of the respective landmark in the mean shape. Using thin plate spline (TPS) tools^32^, we fit the interpolation functions to samples of the landmarks and semi-landmarks cordinates to further visualise the shape variation and observe the exact regions where patterns of variation occur with heatmap. This interpolation refers to the estimation of deformation in the context of shape analysis based on patterns of deformation observed at sample landmarks^33^.

The selected PCs were further subjected to a Discriminant Function Analysis (DFA) to determine the most salient aspects of facial shape for distinguishing the variation in age group^27^. The confusion matrix was computed which is the ability of DFA to assign individuals to the correct pre-defined age group. The GPA, PCA and lollipop graph were computed in MorphoJ 1.06d^34^; EDMA, CVA and MANOVA were performed in PAST 2.17^35^ while DFA was computed in R 5.1^36^.

### Consent for publication

The Angelman Syndrome Foundation (ASF) obtained informed consent from patients or guardians to collect and store images of their faces and make them available in a public repository. We accessed the public repository according to its terms of use. Based on the method by which the subjects were collected from the publicly available source which was acceptable research practice, we do not require special consent from the participants except permission to use the subjects for research, which was granted by the administrative assistant of ASF, Sandy Ruffalo (SRuffalo@angelman.org) and no any image or identity of the participant was revealed in the study according to the agreement.

## Results and discussion

### PCA, CVA and TPS

**Figure 3:**
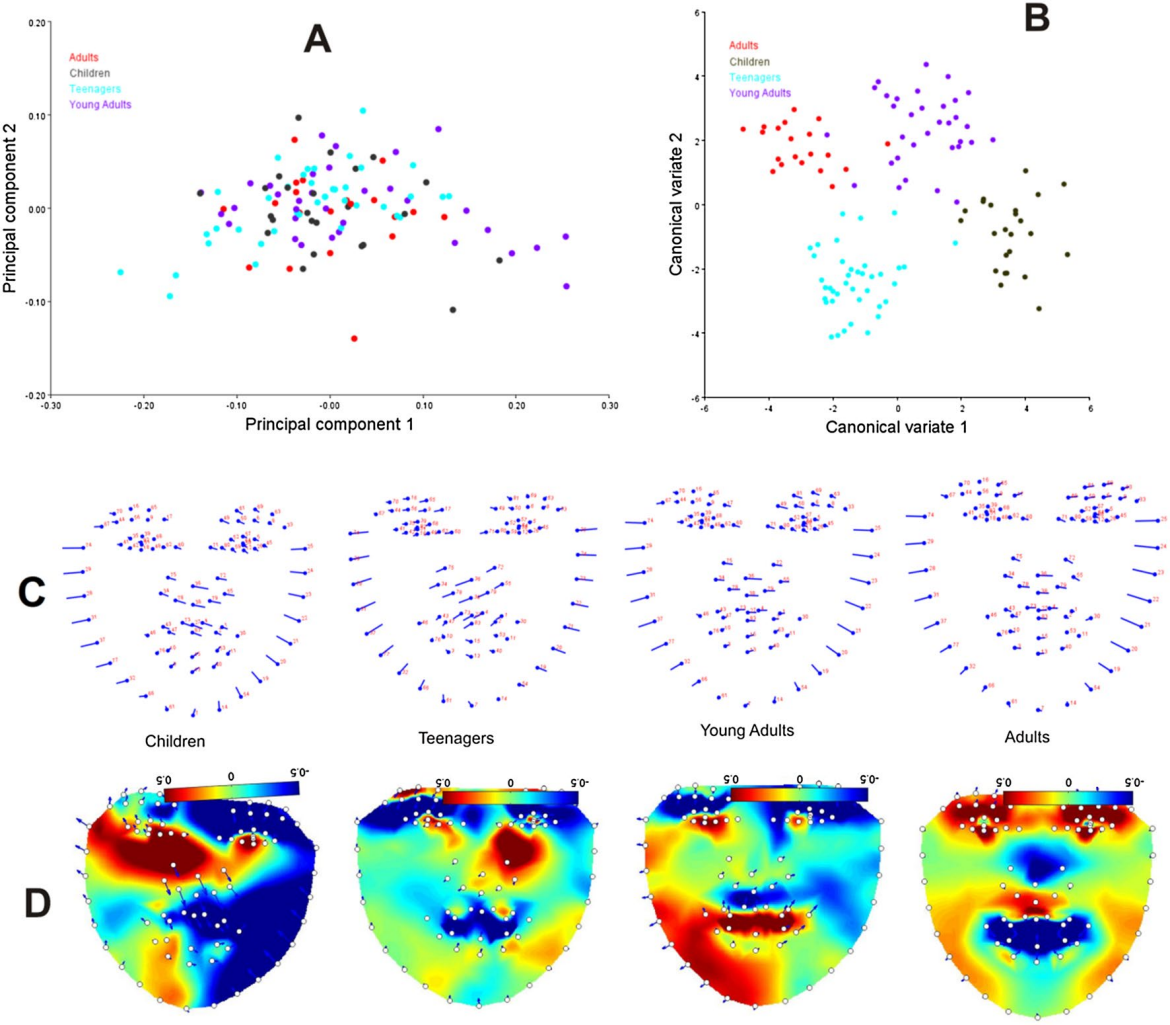
PCA and visualization. (**A**) Distribution of specimen in morphospace along PC1 versus PC2. (**B**) Canonical variate analyses of the first axis plotted against the second axis. (**C**) Lollipop graphs associated with the first PC of shape variability for morphological differences between the average groups. (**D**) Heatmaps of TPS interpolations of local changes in facial regions based on each set of landmarks and semi-landmarks (the software Lori 1.0^32^ was used to process the heatmaps).

For all computed PCs, PC1 explains more than half of the total variation, which indicates that shape variation is concentrated in a single dimension of the shape space^37^. The PCA of the total sample yielded 115PCs, with few zero variability. The first 2PCs accounted for more than 58% of the shape variation (PC1: 53.87%, PC2: 12.14%). The distribution of specimens in morphospace along PC1 to PC2 is shown in Fig. 3A. The PCA of each age group was separately computed: children (PC1: 46.51%, PC2: 22.07%), teenagers (PC1: 60.13%, PC2: 10.99%), young adults (PC1: 68.23%, PC2: 9.11%), and adults (PC1: 38.19%, PC2: 22.33%).

The CVA based on the principal components of the whole dataset achieved strong delimitations (Fig. 3B). Each form well-defined clusters with little overlap in adults and young adults, and the overall MANOVA statistics confirmed that the group means are significantly different using Wilks’ lambda test ($\Lambda_{{{\text{wilks}}}}$ = 0.0856, F = 1.6, *P* = 0.001).

For the sake of visualization, we only presented the deformations of the first PC of each group which accounted for the largest variation using lollipop graphs (Fig. 3C). In the visualization of the specimens, the number of landmarks is shown in red and the mean configuration is shown in light blue. Each of those circles is the average position of the landmarks that are used and the sticks tell us which way things change along with the principal components^38^. If a specimen has a high warp score, then the shape is further down the stick.

In children, the nose and the upper lip protruded rightward and downward while the lower lip sank inward and slightly rightward. The upper region of the left cheek projected outward while from the chin to the upper right cheek projected inward. In teenagers, the nose and the upper lip protruded leftward and downward while the lower lip sank inward and slightly leftward. From the chin to the upper region of the left cheek, there is inward projection while the upper right cheek is projected outward. In young adults, the nose and the upper lip protruded rightward but not downward while the lower lip sank inward and slightly rightward. The upper region of the left cheek projected outward while from the chin to the upper right cheek projected inward. The adults follow the same pattern with young adults, the nose and the upper lip protruded rightward but not downward while the lower lip sank inward and slightly rightward. The upper region of the left cheek projected outward, while the chin to the upper right cheek projected inward. The patterns of shape variation differ between interpolated and landmark data in the facial regions for each age group (Fig. 3D). These visualization results of the heatmap match with the results achieved using relative warps of lollipop graph in Fig. 3C.

### Anthropometric linear distances

The anthropometric measurement results for all age groups are summarized in Table 2; detailed results for all age groups are available as supplementary info S2. In the eye region, the palpebral fissure length is longer in children and shorter in young adults; the intercanthal width and outercanthal width are wider in children followed by teenagers but narrower in young adults and adults. In the face region, children possess a wider facial width, followed by teenagers; while young adults and adults possess the same facial width. In the cheek and chin region, mandibular width is wider in children, followed by young adults and then adults; while chin height is longer in adults and young adults, followed by teenagers and finally children. In the nose region, nasal width is wider in children, followed by teenagers; while equal width is revealed in young adults and adults. The nose is more protruded in children than in other age groups. In the mouth region, labial fissure width is equal and wider in children and teenagers and equal and narrower in young adults and adults; while philtrum length is longer in children than in other age groups.

**Table 2:** Anthropometric measurement for all age groups.

Landmark	Measurement	Children	Teenagers	Young adults	Adults
**Eye region**					
41–62	Palpebral fissure length	1.33	1.30	1.25	1.28
62–80	Intercanthal width	1.48	1.44	1.41	1.42
41–45	Outercanthal width	1.86	1.83	1.80	1.81
**Face region**					
24–29	Facial width	2.06	2.03	2.00	2.00
**Cheek/chin region**					
20–27	Mandibular width	1.68	1.61	1.63	1.62
7–13	Chin height	1.34	1.36	1.37	1.37
**Nose region**					
34–55	Nasal width	1.51	1.48	1.46	1.46
36–55	Nasal protrusion	1.22	1.16	1.18	1.18
**Mouth region**					
30–46	Labial fissure width	1.66	1.66	1.64	1.64
27–38	Philtrum length	1.01	0.92	0.88	0.86
**Total**		**15.16**	**14.78**	**14.63**	**14.64**

The bold text under Landmark column represents the observed facial regions while the bold text under Total row represents the aggregate of each age group.

For each age group, a single discriminant function was derived, indicating that developmental variation could be distinguished based on face shape. The confusion matrix is presented in Table 3. Looking at the cross-validation results, children, young adults, and adults show a similar discriminating patterns (100%). The lowest accuracy observed was in teenagers (96.67%).

**Table 3:** Confusion matrix of percentage classification in age group.

%	Children	Teenagers	Young adults	Adults
Children	100	0	0	0
Teenagers	0	96.67	3.33	0
Young adults	0	0	100	0
Adults	0	0	0	100

To have a clearer understanding of the variations in the shape of the face among the AS patients based on 83 landmarks, a canonical variate analysis was performed. CVA scatter plots revealed differences in face shapes among the age group. Specimens are separated with significantly different group means. Through the lollipop graphs, it was observed that nose, upper and lower lips, right and left cheek, and chin are major contributors in the variation existing among the Angelman syndrome patients. Generally, rightward and downward protrusion of the nose and upper lip and slightly rightward inward sinking of the lower lip are commonly noticed in children, young adults, and adults. No observable changes were detected in the eye region for all groups except in adults.

When measurements were assessed by anatomical region, different ontogenetic patterns of facial traits were apparent. There is a sharp decrease in length from children to adults in palpebral fissure length, intercanthal width, outercanthal width, facial width, nasal width, labial fissure width, and philtrum length. In contrast, there is a steady decline in chin height from adults to children. The infinitesimal differences in shape mapped continuously over entire shape configurations which give rise to shape variables that differ from other variables such as partial warp scores in their interpretation^32^.

DFA is a multivariate data reduction technique that works by constructing a weighted variate optimized to achieve maximum separation between groups^27^. The ability of the discriminant function to correctly assign individuals to their pre-defined group was reported with a classification accuracy of 98.77%. It is however observed that variation in morphological traits is more significant in the children group than in the adults group. Though currently, to the best of our knowledge, no morphological study or anthropometric analysis on Angelman syndrome for age-related variation has been conducted for results comparison. However, it has been pointed out that AS children with uniparental disomy (UPD) exhibited a significant overeating behavior and have better physical growth^6,13^. The studies also confirmed that some weight gain can occur during young adulthood.

Conclusively, influence on facial shape is looked into under the age effect and there is an identification of a clear effect in the analysis. The results demonstrate slight changes in the patterns of shape among the age classes. More so, the statistically significant difference among age groups is found when the face shape is compared per age group and in the distances measured and high variation is noticed in the children group which also matches with the results achieved in the heatmap generated through TPS. Although there is a paucity of studies in morphometrics for investigating shape differences among genetic syndrome subjects, the results reported in this study show that geometric morphometric can provide additional information concerning shape differentiation among taxa that might otherwise be overlooked^39^. However, the landmarks assigned may be inadequate in reflecting the shape of the whole face under study as a result of biological reality reflection uncertainty, which may as well negatively impact the biological variability within the sample related to age. This may require further clarification for reproducibility. In the future, more anatomical landmarks will be assigned and more anthropometric measurement will be carried out to increase the variability and significance of the study.

### Ethics

By human subjects ethics, the method by which the dataset was collected from the publicly available source was acceptable research practice and does not require special consent from the participants or a Research Ethics Committee. But advice from research ethics board members and legal services were sought in arriving at this conclusion; though permission to use the subjects was granted by the administrative assistant of ASF.

## Supplementary Information

Supplementary Information 1.

Supplementary Information 2.

Supplementary Information 3.
