# Supplementary material for: Investigation of age-related facial variation among Angelman syndrome patients
Source: Sci Rep. 2021 Oct 21;11:20767. doi: 10.1038/s41598-021-99944-z (PMC8531312; doi:10.1038/s41598-021-99944-z)
Supplement: Supplementary file 1 — Supplementary Information 1. [file 41598_2021_99944_MOESM1_ESM.docx]

**Investigation of Age-related Facial variation among Angelman Syndrome Patients**

Olalekan Agbolade^1*^ , Azree Nazri^1*^, Razali Yaakob^1^, Abdul Azim Ghani^2^, Yoke Kqueen Cheah^3^

Supplementary info S1: Two-dimensional raw data for each age group in Angelman syndrome.

Supplementary info S2: Anthropometric linear distances by EDMA for each age group.
